# The Value of Adjunctive Left Atrial Posterior Wall Isolation on Clinical Outcomes in Atrial Fibrillation Patients: A Systematic Review and Meta-Analysis

**DOI:** 10.31083/j.rcm2506210

**Published:** 2024-06-05

**Authors:** Lianfeng Liu, Yu Geng, Yuanwei Liu, Tingting Lv, Ping Zhang

**Affiliations:** ^1^Department of Cardiology, Beijing Tsinghua Changgung Hospital, School of Clinical Medicine, Tsinghua University, 100084 Beijing, China

**Keywords:** atrial fibrillation, left atrial posterior wall isolation, pulmonary vein isolation

## Abstract

**Background:**

Although pulmonary vein isolation (PVI) remains the mainstream way of atrial fibrillation (AF) ablation. The left atrial posterior wall (LAPW) may contributes to the development of AF as an arrhythmogenic substrate. The efficacy of additional left atrial posterior wall isolation (LAPWI) beyond PVI is in AF patients remains undefined. This study explored the influence of posterior wall isolation (PWI) on clinical outcomes in AF patients.

**Methods:**

PubMed, EMBASE, and Cochrane Library databases were searched for studies comparing the outcomes of AF with and without PWI. The efficacy outcomes were recurrence of all atrial arrhythmia (AA), atrial fibrillation (AF), and atrial flutter (AFL)/atrial tachycardia (AT). The safety outcomes were mainly focused on procedural adverse events.

**Results:**

A total of 16 studies (7 randomized controlled trials (RCTs), 3 prospective studies and 6 retrospective analyses) with 3340 AF patients were enrolled (1550 patients in PVI with PWI group and 1790 in PVI alone group). 12 studies included persistent atrial fibrillation patients, 3 studies with paroxysmal AF patients and 1 study with paroxysmal AF and persistent AF concurrently. Mean follow-up period was 16.56 months. In AF patients, adjunctive PWI obviously reduced the recurrence of all atrial arrhythmias (risk ratio (RR) 0.78 [95% CI 0.64–0.95], $I^{2}$ = 79%, *p* = 0.01) and the recurrence of AF (RR 0.68 [95% CI 0.53–0.88], $I^{2}$ = 75%, *p* = 0.004); Meanwhile, additional PWI left no impact substantially on lower recurrence of AFL/AT (RR 1.23 [95% CI 0.94–1.60], $I^{2}$ = 49%, *p* = 0.12). The results seemed to be no significant differences in occurrence rate of procedural complications between the PVI only and PWI+PVI (RR 1.19 [95% CI 0.80–1.79], $I^{2}$ = 0%, *p* = 0.39). In subgroup analyses, the benefit of adjunctive PWI compared with PVI only was more distinct in persistent AF group and cryoballoon ablation group. Notably, adjunctive PWI with radiofrequency ablation may induce a slight increase of recurrent AFL/AT compared with PVI only (RR 1.56 [95% CI 1.02–2.39], $I^{2}$ = 30%, *p* = 0.04).

**Conclusions:**

Compared with PVI alone, additional PWI to PVI appeared to be associated with decreased recurrence of AF and atrial arrhythmias without an increased occurrence of procedural complications, especially in persistent AF patients. Cryoballoon ablation seemed more suitable for PWI compared with radiofrequency ablation. More RCTs are needed to verify the conclusion.

## 1. Introduction

Atrial fibrillation (AF) is a global issue and the most diagnosed supraventricular arrhythmia in adults, affecting approximately 43.6 million individuals globally. It is associated with a series of cardiovascular and cerebrovascular diseases and high mortality [1, 2]. Catheter ablation has emerged as an effective rhythm control strategy for AF, demonstrating benefits in improving long-term prognosis and reducing adverse events in symptomatic AF patients, as supported by previous research [3, 4, 5, 6]. Recent guidelines have endorsed catheter ablation as a first-line therapy and superior alternative to antiarrhythmic drugs (AADs) for maintaining sinus rhythm and improving symptoms in select patient populations [2].

Generally, pulmonary vein isolation (PVI) is considered the basic and routine ablation strategy widely used for AF treatment [2]. But it is regrettable that the sinus rhythm maintenance rate after PVI remains relatively low, especially in persistent AF (PerAF) patients [7, 8]. Left atrial posterior wall isolation (PWI), as an extensive ablation procedure, has been proposed in addition to PVI to target non-pulmonary vein triggers [2]. Previous trials have suggested that adjunctive PWI may yield better clinical outcomes compared to PVI alone [9, 10]. However, the CAPLA study (Catheter ablation for persistent atrial fibrillation: pulmonary vein isolation (PVI) versus PVI with posterior left atrial wall isolation (PWI)) reported that adding PWI to PVI appeared no significant improvement in outcomes when compared to PVI alone [11]. The efficacy of adjunctive PWI remains a topic of debate. Given this knowledge gap, we conducted a systematic meta-analysis with the latest clinical studies included, encompassing both randomized controlled trials (RCTs) and non-RCTs, to provide insights into the role of adjunctive PWI in AF patients.

## 2. Materials and Methods

### 2.1 Search Strategy and Data Extraction

We executed the meta-analysis in line with the Preferred Reporting Items for Systematic Reviews and Meta-analyses (PRISMA) guidelines (as seen in **Supplementary Table 1**) [12]. Our study protocol has been officially registered on the International Platform of Registered Systematic Review and Meta-analysis Protocols (INPLASY202380127). The full document can be accessed at inplasy.com (https://inplasy.com/inplasy-2023-8-0127).

A comprehensive search was undertaken in PubMed, EMBASE, and the Cochrane Central Register of Clinical Trials (CENTRAL) for researches that assessed the efficacy of adjunctive posterior wall isolation on the clinical prognosis of patients with atrial fibrillation up until August 30, 2023. We did not limit our search by language. To augment this search, we also performed a manual search of reference lists from pertinent articles and guidelines issued by professional societies. The details of literature search strategy can be reached in the supplement (**Supplementary Table 2**). Studies enrolled should meet the following criteria: (1) Patients with atrial fibrillation. (2) Additional left atrial posterior wall isolation (LAPWI) beyond PVI or PVI only was performed of these patients. (3) Outcomes Indicators: recurrence of all atrial arrhythmia (AA), AF, atrial flutter (AFL)/atrial tachycardia (AT) and procedural adverse events, including one.

The protocol was carefully crafted by two authors (LFL and YG) and subsequently reviewed by all co-authors. For document management, we employed the EndNote software Version X9 (Clarivate Analytics, London, United Kingdom). Two investigators (LFL and YG) independently assessed the suitability of the identified studies, with any disagreements being resolved through discussion with the senior author (PZ).

### 2.2 Outcomes

The primary outcome encompassed the recurrence of all forms of atrial arrhythmia, atrial fibrillation, atrial flutter/atrial tachycardia. Secondary outcomes targeted was a composite endpoint of safety outcomes, mainly including procedural adverse events. We reviewed definitions employed in individual trials and aimed to use a consistent definition across trials wherever feasible (Refer to **Supplementary Table 3**). The Cochrane Collaboration criteria were utilized to assess the risk of bias in each incorporated study [13].

### 2.3 Statistical Analysis

The meta-analysis was conducted using Revman5.3 (The Nordic Cochrane Center, Copenhagen, Denmark). For data that exhibited homogeneity (*p* > 0.10 and $I^{2}$ ≤ 50%), a fixed-effect model was applied for the meta-analysis. In cases where homogeneity was not achieved (*p* > 0.10 and $I^{2}$ ≤ 50%), and heterogeneity could not be disregarded, a random-effects model was utilized for combining effects [14]. Notably, sensitivity and subgroup analyses should be contemplated when analyzing this type of data. For continuous outcomes, we estimated the mean differences (MD) and the associated 95% confidence intervals (CIs). Several RCTs presented the median as the measure of treatment efficacy, along with the interquartile range (IQR). In such cases, we deduced the mean from the median and estimated standard deviations (SDs) from the IQR using methodologies from earlier research [15]. A *p*-value of <0.05 was deemed statistically significant.

### 2.4 Subgroup and Sensitivity Analyses

The therapeutic efficacy and safety of adjunctive LAPWI beyond PVI as compared to PVI only were scrutinized in AF patients. However, the type of atrial fibrillation and the ablation energy differed among the studies. Supplementary subgroup analyses were carried out to compare the efficacy and safety between the paroxysmal AF patient group and the persistent AF patient group, and between the radiofrequency ablation group and the cryoballoon ablation group. Revman was employed to determine the impact of individual studies on the aggregate pooled estimate for each predefined outcome.

## 3. Results

**Fig. 1.**
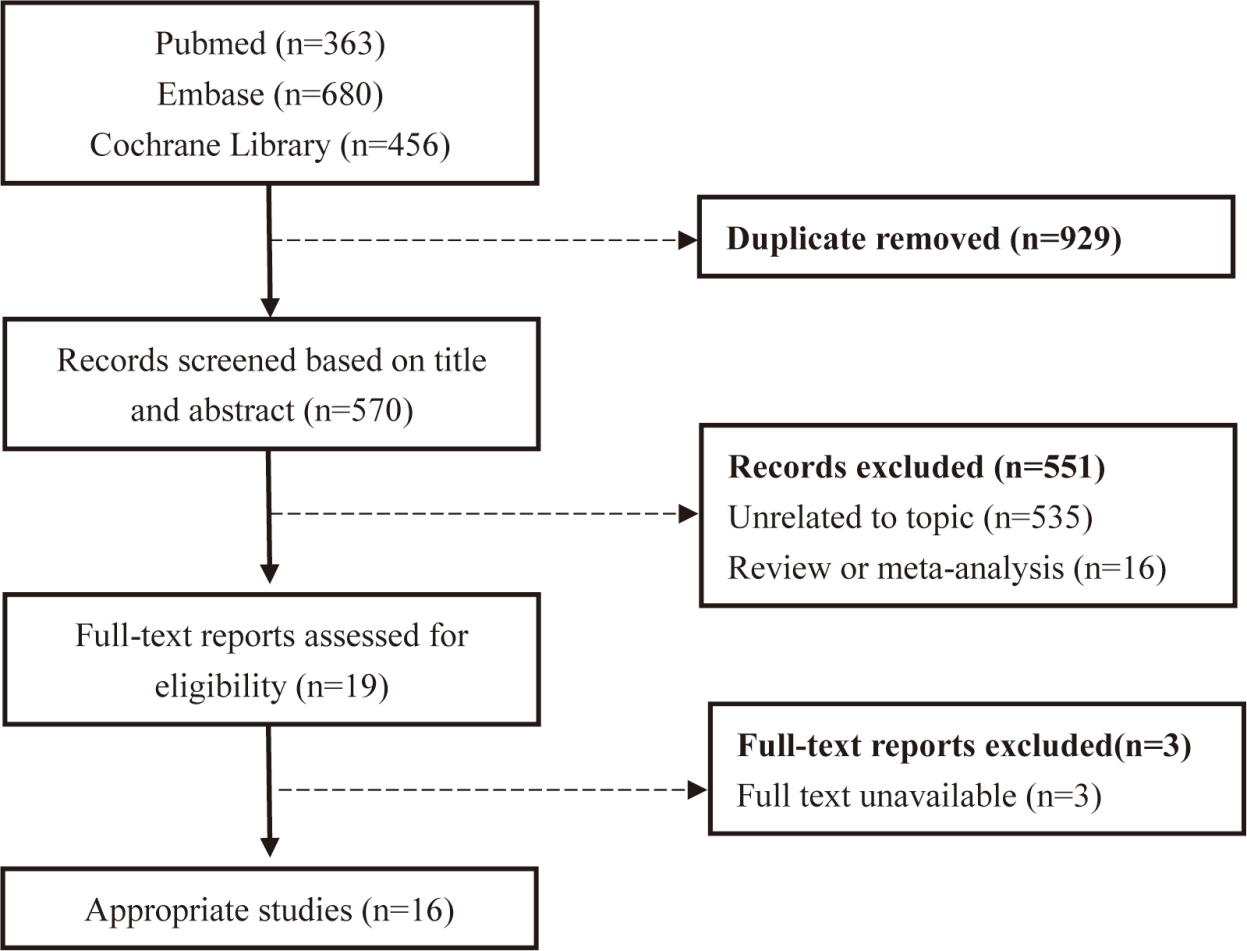
**Flow diagram of the study selection process**.

The study selection process is summarized in the flow chart (Fig. 1). We identified a total of 1499 studies from our search across PubMed, Cochrane Central Register for Controlled Trials, and EMBASE. Among these, 929 were excluded due to duplication. Further scrutiny of titles and abstracts led to the exclusion of another 551 studies. After a full-text review of the remaining 19 studies, 16 trials that compared the efficiency and safety of adjunctive PWI to PVI in AF patients were selected for inclusion.

Ultimately, the analysis encompassed 16 trials with 3340 patients: 1550 patients were allocated to the PVI with PWI group and 1790 to the PVI alone group (Tables 1,2, Ref. [9, 10, 11, 16, 17, 18, 19, 20, 21, 22, 23, 24, 25, 26, 27, 28]). Table 1 summarizes patient baseline characteristics, and Table 2 describes procedure characteristics. Among them, Jankelson *et al*. [16], Bisignani *et al*. [17] and Aryana *et al*. [18] included only paroxysmal AF patients (totally 721 patients enrolled). Kim *et al*. [19] simultaneously enrolled paroxysmal and persistent AF patients (91 patients included was paroxysmal atrial fibrillation (PAF) with 53% in PVI group). The others left contained only persistent AF patients (totally 2469 patients enrolled). When it comes to the ablation method, those trials applying cryoballoon ablation as the main ablation energy included Aryana *et al*. [10], Ahn *et al*. [9], Bisignani *et al*. [17], Aryana *et al*. [20], Nishimura *et al*. [21] and Aryana *et al*. [18] (totally 1100 patients included). The rest mainly with radiofrequency ablation. The mean follow-up time was about 16.56 months. There were no differences observed in two groups in terms of the proportion of patients lost to follow up across trials.

**Table 1.** **Patient baseline characteristics in the meta-analysis**.

Author/year	Design type	AF type	Total patients		Female, n (%)		Mean age		LAD mm	
			PVI	PVI+PWI	PVI	PVI+PWI	PVI	PVI+PWI	PVI	PVI+PWI
Aryana 2021 [10]	RCT	PerAF	55	55	22 (40)	20 (36)	70 ± 9	67 ± 8	44 ± 5	44 ± 4
Kistler 2023 [11]	RCT	PerAF	168	170	40 (23.8)	39 (22.9)	65.5 (57.8–71.7)	65.7 (58.7–71.1)	44 ± 7	46 ± 0.6
Kim 2015 [23]	RCT	PerAF	60	60	19 (31.7)	60 (23.3)	58.3 ± 9.6	56.2 ± 11.9	42.1 ± 5.1	42.3 ± 6.4
Ahn 2022 [9]	RCT	PerAF	50	50	5 (10)	11 (22)	65.9 ± 8.8	65.1 ± 8.6	48.5 ± 8.1	48.1 ± 7.4
Murata 2022 [24]	Prospective study	PerAF	212	212	71 (20.1)	58 (26.6)	64.3 ± 10.2	67.7 ± 9.5	42.3 + 6.2	42.2 + 7.0
Pak 2020 [25]	RCT	PerAF	57	57	17 (29.8)	15 (26.3)	61.6 ± 7.8	58.6 ± 11.4	42.7 ± 6.1	41.4 ± 6.1
Kim 2022 [19]	RCT	PAF+PerAF	75	75	13 (17.3)	22 (29.3)	59.1 ± 9.9	60.0 ± 9.9	41.0 ± 5.8	41.7 ± 7.1
Tokioka 2021 [26]	retrospective study	PerAF	91	90	19 (20.9)	23 (25.6)	66.9 ± 10.6	67 ± 9.9	42.5 ± 7.1	42.4 ± 6.2
Jankelson 2022 [16]	retrospective study	PAF	214	107	69 (32.2)	38 (35.5)	60.7 ± 11.7	63.3 ± 11	27 (23, 31)	26 (21, 31)
Bisignani 2020 [17]	retrospective study	PAF	50	30	21 (42)	10 (33.3)	67.4 ± 8.5	68.4 ± 9.16	43.9 ± 9.5	44.8 ± 4.7
Aryana 2018 [20]	prospective study	PerAF	168	222	60 (36)	76 (34)	67 ± 11	67 ± 9	44 ± 7	47 ± 9
Bai 2016 [27]	prospective study	PerAF	20	32	4 (20)	5 (16)	63 ± 11	64 ± 10	49 ± 4	48 ± 7
Sutter 2019 [22]	retrospective study	PerAF	255	78	84 (33)	25 (32)	67 (59–73)	66 (56–74)	42 ± 7	46 ± 7
Nishimura 2019 [21]	retrospective study	PerAF	50	50	8 (16)	8 (16)	66 ± 10	62 ± 10	43 ± 6	44 ± 5
Lee 2019 [28]	RCT	PerAF	105	102	21 (20)	14 (13.7)	58.6 ± 11	58.9 ± 10.5	44.5 ± 6.7	45 ± 5.3
Aryana 2023 [18]	retrospective study	PAF	160	160	54 (34)	61 (38)	63 ± 11	63 ± 10	44 ± 7	44 ± 5

RCT, randomized controlled trial; AF, atrial fibrillation; PVI, pulmonary vein isolation; PWI, posterior left atrial wall isolation; LAD, left ventricular posterior; PAF, paroxysmal atrial fibrillation; PerAF, persistent atrial fibrillation.

**Table 2.** **Patient procedure characteristics in the meta-analysis**.

Author/year	Main ablation	Procedure time		Ablation time		Fluoroscopy time		Follow-up
		PVI	PVI+PWI	PVI	PVI+PWI	PVI	PVI+PWI	
Aryana 2021 [10]	CBA	127 ± 40	168 ± 34	29 ± 14	51 ± 15	NR	NR	12
Kistler 2023 [11]	RF	120.5 ± 56.8	142 ± 69.3	28 ± 11.7	34.2 ± 20.7	10.5 ± 7.2	11.5 ± 6.8	12
Kim 2015 [23]	RF	154.9 ± 57.1	163.1 ± 47.2	121.7 ± 58.7	128.9 ± 37.9	NR	NR	12
Ahn 2022 [9]	CBA	74.3 ± 8.9	98.8 ± 6.2	25.8 ± 3.1	38.8 ± 5.1	4.6 ± 2.6	6.1 ± 4.7	12
Murata 2022 [24]	RF	NR	NR	NR	NR	NR	NR	12
Pak 2020 [25]	RF	179.1 ± 60.2	186.2 ± 52.7	69.79 ± 15.87	88.94 ± 25.28	NR	NR	23.8 ± 10.2
Kim 2022 [19]	RF	101 (86–121)	120 (100–138)	18.06 (13.23–27.73)	26.58 (20.73–38.37)	NR	NR	17
Tokioka 2021 [26]	RF	NR	NR	NR	NR	NR	NR	19
Jankelson 2022 [16]	RF	128 ± 29	125 ± 28	26.7 ± 8.7	28.6 ± 10.4	12.3 ± 7.8	14.8 ± 7.4	12
Bisignani 2020 [17]	CBA	58.42 ± 16.62	93.56 ± 21.98	NR	NR	14.7 ± 7.4	25.23 ± 13	12
Aryana 2018 [20]	CBA	97 ± 29	188 ± 42	Not reported	Not reported	19 ± 7	28 ± 9	12
Bai 2016 [27]	RF	204 ± 96	216 ± 66	74 ± 40	88 ± 41	58 ± 29	62 ± 26	36
Sutter 2019 [22]	RF	NR	NR	52 ± 28	53 ± 21	NR	NR	6
Nishimura 2019 [21]	CBA	192 ± 33	153 ± 52	NR	NR	NR	NR	12
Lee 2019 [28]	RF	206.8 ± 77.7	226.7 ± 63.1	71.48 ± 30.61	89.41 ± 39.3	35 ± 18.2	38 ± 16.6	16.2 ± 8.8
Aryana 2023 [18]	CBA	103 ± 24	127 ± 14	23 ± 10	42 ± 11	13 ± 8	20 ± 6	39 ± 9

PVI, pulmonary vein isolation; PWI, posterior left atrial wall isolation; RF, radiofrequency ablation; CBA, cryoballoon ablation; NR, no record.

### 3.1 Clinical Outcomes

**Fig. 2.**
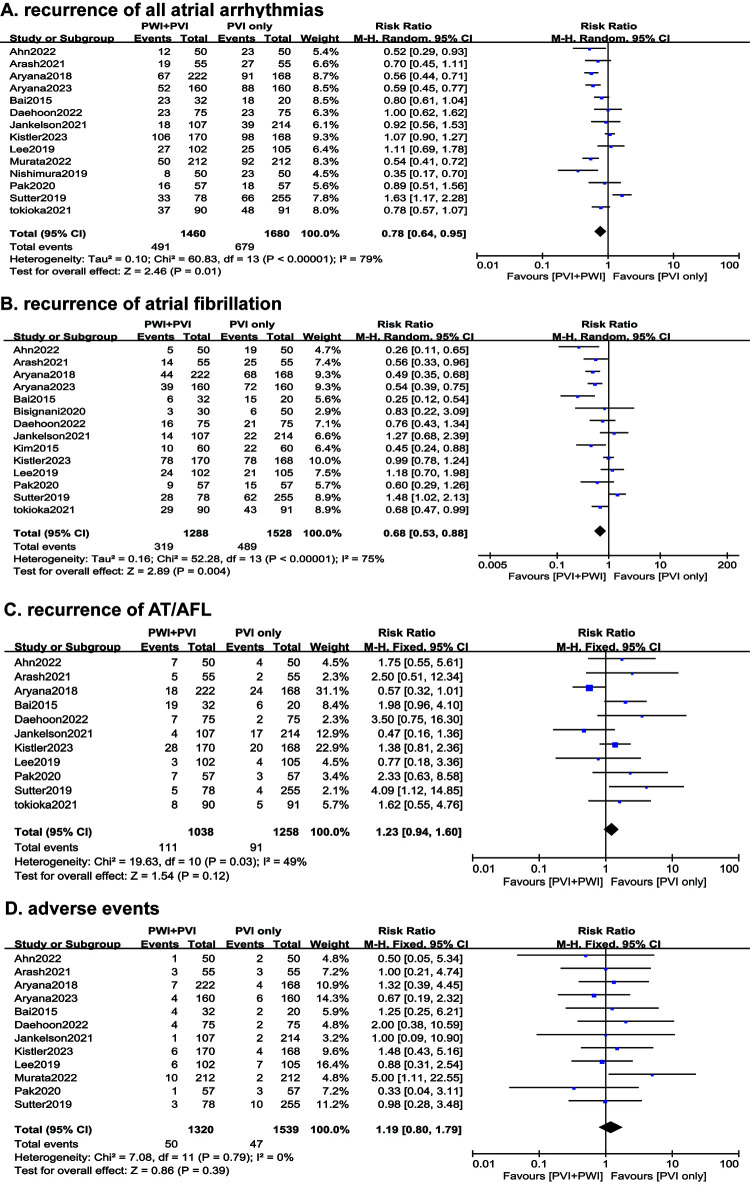
**Comparisons of the outcomes between the patients assigned to PVI with PWI and PVI alone. **(A) The recurrence of all arrhythmias. (B) The recurrence of AF. (C) The recurrence of AT/AFL. (D) The adverse events. AF, atrial fibrillation; PWI, posterior wall isolation; PVI, pulmonary vein isolation; AT, atrial tachycardia; AFL, atrial flutter.

Patients assigned to the PVI with PWI group, when compared to the PVI alone group, demonstrated reduced odds of the recurrence of AA (risk ratio (RR) 0.78 [95% CI 0.64–0.95], $I^{2}$ = 79%, *p* = 0.01) (Fig. 2A). This was especially evident in the recurrence of AF (RR 0.68 [95% CI 0.53–0.88], $I^{2}$ = 75%, *p* = 0.004) (Fig. 2B). However, no significant differences were observed between the groups in the recurrence odds of AT/AFL (RR 1.23 [95% CI 0.94–1.60], $I^{2}$ = 49%, *p* = 0.12) (Fig. 2C). Similarly, the odds of adverse events (RR 1.19 [95% CI 0.80–1.79], $I^{2}$ = 0%, *p* = 0.39) revealed no distinct difference between the two groups (Fig. 2D).

### 3.2 Subgroup Analysis

We conducted a predetermined subgroup analysis based on the type of AF. Of the patients, 721 had paroxysmal AF (297 in the PVI with PWI group and 424 in the PVI alone group), while 1945 had persistent AF (916 in the PVI with PWI group and 1029 in the PVI alone group).

There was no notable difference in odds of the recurrence of AA (RR 0.71 [95% CI 0.45–1.12], $I^{2}$ = 62%, *p* = 0.14) and AF recurrence (RR 0.79 [95% CI 0.41–1.50], $I^{2}$ = 65%, *p* = 0.47) for paroxysmal AF patients between the groups. However, for persistent AF patients, the PVI with PWI group showed decreased odds of the incidence of recurrent of AA (RR 0.77 [95% CI 0.61–0.98], $I^{2}$ = 82%, *p* = 0.03) and AF recurrence (RR 0.65 [95% CI 0.47–0.90], $I^{2}$ = 80%, *p* = 0.009) (**Supplementary Fig. 1**). As to the incidence of the recurrent AFL/AT and procedural complications, the additional PWI to PVI made no difference in both PAF and PerAF patients.

Furthermore, we conducted a subgroup analysis based on the ablation energy. Of the patients, 1020 underwent cryoballoon ablation (537 in the PVI with PWI group and 483 in the PVI alone group), while 2120 underwent radiofrequency ablation (923 in the PVI with PWI group and 1197 in the PVI alone group). The cryoballoon ablation patients in the PVI with PWI group showed reduced odds of the recurrence of AA (RR 0.57 [95% CI 0.49–0.66], $I^{2}$ = 0%, *p* < 0.001) and AF recurrence (RR 0.51 [95% CI 0.42–0.62], $I^{2}$ = 0%, *p* < 0.001). However, no obvious distinctions in odds were observed among the radiofrequency ablation patients between the groups for the incidence of recurrent of AA (RR 0.93 [95% CI 0.75–1.15], $I^{2}$ = 74%, *p* = 0.49) and AF recurrence (RR 0.81 [95% CI 0.60–1.09], $I^{2}$ = 72%, *p* = 0.16) (**Supplementary Fig. 2**). Interestingly, a mild increase of the incidence of recurrent AFL/AT happened in the radiofrequency ablation patients with adjunctive PWI (RR 1.56 [95% CI 1.02–2.39], $I^{2}$ = 30%, *p* = 0.04), while no obvious distinctions in cryoballoon ablation subgroup (RR 1.12 [95% CI 0.42–2.98], $I^{2}$ = 61%, *p* = 0.82). Both in cryoballoon and radiofrequency groups, adjunctive PWI after PVI did not affect the safety outcomes.

We simultaneously conduct the analysis of efficacy and safety endpoints in RCTs and non-RCTs to focus on the results of RCTs. 7 trials belongs to RCTs (569 in the PVI with PWI group and 570 in the PVI alone group), while 9 trials belongs to non-RCTs (981 in the PVI with PWI group and 1220 in the PVI alone group). In RCTs, similar to the results of overall studies analysis, when compared to the PVI alone group, adjunctive PWI group demonstrated reduced odds of the recurrence of AF (RR 0.68 [95% CI 0.49–0.96], $I^{2}$ = 64%, *p* = 0.03). But no obvious differences were observed between the groups in the recurrence odds of AA (RR 0.91 [95% CI 0.73–1.13], $I^{2}$ = 39%, *p* = 0.38). In contrast, a obvious increase of the incidence of recurrent AFL/AT happened in the adjunctive PWI group (RR 1.59 [95% CI 1.06–2.38], $I^{2}$ = 0%, *p* = 0.03). At Last, the odds of adverse events (RR 1.01 [95% CI 0.56–1.82], $I^{2}$ = 0%, *p* = 0.98) revealed no distinct difference between the two groups (**Supplementary Fig. 3**).

### 3.3 Sensitivity Analysis 

Utilizing Revman, we assessed the impact from each individual study on the overall pooled estimates for each predefined outcome. Our findings suggest that the exclusion of any specific study would not significantly alter the results. Likewise, there were no substantial differences in other efficacy and safety outcomes. Sensitivity analysis using the Mantel-Haenszel fixed effects models with RR as the effect measure produced congruent findings (**Supplementary Fig. 4**).

### 3.4 Risk of Bias and Quality Assessment of Outcomes

The results about the risk of bias assessment for randomized control trials with the revised Cochrane risk of bias tool for randomized trials (RoB2) are summarized in **Supplementary Fig. 5**. Six studies were considered at low risk for overall risk of bias.

## 4. Discussion

As far as we know, this is the most recent systematic review and meta-analysis of RCTs and non-RCTs investigating the overall efficacy of PWI as an adjunctive strategy in catheter ablation for AF patients. Our research results indicated that the addition of PWI to PVI led to a lower incidence of recurrent AF and AA in AF patients compared to PVI alone. Meanwhile, PVI combined with PWI had no relationship with any extra benefit in lowering the recurrence of atrial flutter or atrial tachycardia. Compared with the PVI alone group, the adjunctive PWI group seemed not to induce obvious difference in adverse events, in terms of safety endpoints. Subgroup analysis indicated that the reduction in the recurrence of AF and AA was more significant in subgroups of persistent AF and cryoballoon ablation (CBA) compared to paroxysmal AF and radiofrequency ablation (RFA) groups. Similar to the main findings, no differences in adverse event incidence were observed when stratified by AF type or ablation strategy. Interestingly, the recurrence of atrial flutter/atrial tachycardia seemed to be higher in RFA group than that in the CBA group. In addition, the incidence of atrial flutter/atrial tachycardia after ablation was similar between the persistent AF and paroxysmal AF groups. In RCT trials, the results regarding the recurrence of AF and adverse event incidence were similar with the main findings. But the recurrence of atrial flutter/atrial tachycardia seemed to be higher with adjunctive PWI and no differences in the recurrence of AA when only RCTs enrolled in analysis.

### 4.1 Role of the Left Atrial Posterior Wall in Atrial Fibrillation

Catheter ablation is considered a secure and efficient alternative for maintaining sinus rhythm and improving symptoms in AF patients, when performed by experienced operators. PVI is commonly recommended as the primary ablation strategy for AF [2]. However, the efficacy of PVI alone is insufficient for achieving optimal therapeutic effect, particularly in PerAF and long-standing PerAF patients [2, 29]. A meta-analysis reported a 12-month arrhythmia-free survival rate of only 66.7% when PVI alone was used as a single procedure in PerAF and long-standing PerAF patients [8]. Consequently, adjunctive PWI to PVI has been advocated as a strategy to enhance clinical outcomes in AF patients [30, 31].

Previous studies have demonstrated the existence of AF triggers outside the pulmonary veins (PVs) [32, 33]. When PAF evolves to PerAF or long-standing PerAF, non-PV locations become more relevant, particularly in the posterior wall [34]. The left atrial posterior wall has been identified as an important source of non-PV triggers, with 38% of that originating from posterior wall (PW) region [32, 35]. Embryologically, anatomically, and electrophysiologically, the left atrial posterior wall (LAPW) is closely related to the PVs and shares a common tissue origin [36]. This connection allows the LAPW to serve as a site for AF triggers. Myocytes in the LAPW contribute to sustaining AF due to their electrophysiological properties, characterized by short action potential duration, the shortest refractory period, and a low resting membrane potential [37]. Furthermore, differences in the orientation of myocardial fibers in the LAPW compared to the PVs can lead to local re-entry circuits [38]. Additionally, the presence of ganglionated plexi, which are abundant in the left atrial posterior wall, may play a crucial part in initiation and sustainability of AF [39].

As described above, the LAPW is taken for a crucial substrate in the initiation and maintenance of AF. Based on this, the additional posterior wall ablation adjunctive to PVI may improve the clinical outcomes of AF, particularly lower the reappearance of atrial arrhythmias, which is in keeping with the findings of our research. However, the value of adjunctive PWI in improving AF prognosis remains a topic of debate. Recent meta-analyses have shown that adjunctive PWI is in connection with decreased reappearence of AF and atrial arrhythmias after ablation, without an increased risk of post-procedure atrial flutter/atrial tachycardia, particularly in persistent AF patients [40, 41, 42, 43]. However, the CAPLA study, the most recent RCT, reported that adding PWI to PVI alone did not significantly reduce the recurrence of atrial arrhythmias after 12 months compared to PVI alone after the first-time catheter ablation in Persistent AF patients. Kistler *et al*. [11] suggested that not all persistent AF patients may benefit from adjunctive PWI, and certain patient subgroups with specific characteristics, such as low voltage or longer-standing AF, may experience greater benefits. Further trials, especially RCTs, are needed to determine which patient groups and endpoints are most suitable for adjunctive PWI.

### 4.2 Role of Adjunctive PWI in Paroxysmal and Persistent AF

According to the results of our subgroup analysis, PWI can reduce the recurrence of AF or atrial arrhythmia in persistent AF, but it did not show any extra benefit when adjunctive PWI to PVI in paroxysmal AF. These retrospective analyses related to paroxysmal AF were included. Jankelson *et al*. [16] and Bisignani *et al*. [17] held the idea that additional PWI to PVI in paroxysmal AF patients after ablation did not reduce the recurrence of atrial arrhythmia. Bisignani *et al*. [17] mentioned that using CBA might induce a comparative wide lesion around the PVs, which often extended beyond the antrum and comprised a large section of the LAPW, such situation might explain the negative result. Besides that, non-PV foci appear to be distributed in different areas located at both atria, not much in LAPW. Only 0.13% triggered PAF could be attributed to non-PV foci located at the LAPW [44]. While the IMPPROVE-PAF Trial (Cryoballoon Isolation of Combined Posterior Wall and Pulmonary Veins Versus Pulmonary Veins Alone for the Treatment of Paroxysmal Atrial Fibrillation(NCT05296824)) [18] concluded that, adjunctive PWI could bring PAF patients with more significant freedom from recurrent atrial arrhythmias and AF, which conducted with cryoballoon ablation for a long-term follow-up. They emphasized that recurrences of atrial arrhythmia in PAF patients seem to be charactered with a lower incidence and more asymptomatic than that in PerAF ones after catheter ablation, which might be used to explained why results were different from Bisignani *et al*. [17]. Actually, Mohanty *et al*. [45] revealed that the explanation of late AF recurrence in PAF patients after endurable PVI was almost PV-independent and more reasons attributed to extra-PV triggers. Thus, the understandings towards the efficacy of adjunctive PWI to PAF patients remain poorly understood and controversial. More studies regarding larger sample sizes, longer follow-up durations, or more sensitive or vigorous monitoring equipment may be needed.

Not only our meta-analysis results but also recent studies and meta-analyses gradually confirmed the value of the additional PWI to PVI on improving the therapeutic effect of persistent AF patients, though CAPLA study did not support the empirical deployment of PWI for first-time AF ablation [11]. However, Kistler *et al*. [11] mentioned that approach to rhythm monitoring after ablation, especially implantable devices for surveillance, was important and ideal. Meanwhile, it was needed to identify patient subgroups who may benefit better from adjunctive PWI [11]. Multivariable regression analysis from one large secondary analysis [40] indicated that factors of older age, larger left atrial diameters (LADs), and persistent AF could induced more obvious efficacy of adjunctive PWI in decreasing the arrhythmia recurrence, which can to some extent indicate how to choose proper patients to conduct adjunctive PWI regarding AF as a progressive and age-related diseases.

### 4.3 Role of Adjunctive PWI in Cryoballoon and Radiofrequency Catheter

According to the present meta-analysis, when using CBA, PVI combined with PWI leaded to a reduced recurrence of AF and AA than that in RFA group. Interestingly, adjunctive PWI slightly increased the recurrence of AFL/AT in RFA group when compared with PVI only. As to safety outcomes, no obvious distinction was found between the two subgroups in terms of ablation energy.

Regarding to the results mentioned above, the posterior wall ablated by CBA seemed to lower the recurrence of AF or AA more effectively than RFA. It might be explained by the theory that ablation lesions created by cryoballoon are always wide and durable compared with those by point-by-point radiofrequency catheter [46]. The CONVERGE trial (Convergence of Epicardial and Endocardial RF Ablation for the Treatment of Symptomatic Persistent AF) also showing improved effectiveness with the novel epicardial-endocardial ablation approach compared to endocardial catheter ablation and the importance of the creation of durable lesions inside LAPW [47]. Two RCT trials conducting CBA for adjunctive PWI were enrolled in our analysis [9, 10]. Ahn *et al*. [9], the first RCT to confirm the efficacy and safety of adjunctive PWI employing CBA alone without additional RFA in PerAF patients, stated that CBA strategy could achieve durable PWI by delivering direct cryoenergy on the entire LAPW, so that the isolation of arrhythmogenic substrate, for example ganglionated plexi, could be conducted efficiently. As a contrast, using radiofrequency catheter ablation (RFCA) to achieve PWI must conduct the roof and inferior linear ablations. Therefore, it could be explained why the benefit of PWI was deficient among studies employing RFCA [9].

Particularly worth mentioning, in RFCA subgroup, adjunctive PWI slightly increased the recurrence of AFL/AT, which left some effect on the result of the recurrence of AA to a certain extent. Same to the results of our analysis, Sutter *et al*. [22] included in the present meta-analysis and another study by Yokokawa *et al*. [48] both mentioned an increased occurrence rate of AFL/AT after PWI plus to PVI. A macro-reentrant pathway may be created by isolating a larger area of the atrium, which might be suitable substrate for atrial flutter. Meanwhile, lowering recurrence of AF may create opportunities for maintenance of stable focal AT. This may explain the increase of recurrence of AT/AFL after PWI plus to PVI, especially when the incomplete linear block in PWI happened using the point-by-point fashion in RFCA [22, 49].

However, durable PWI usually means longer procedure time or high-power output, which may arose concerns about the increase in the risk of adverse events related with procedures like phrenic nerve injury or even esophageal damage. Though results from our meta-analysis and recent RCT trials did not show significant difference in the aspect of the incidence of adverse events when considering using the CBA method. Meanwhile, the results indicated that using CBA to achieve PWI could decrease the recurrence of AF/AA in AF patients. But it is still hard to conclude that CBA is prior to RFCA. Previous trials revealed that CBA was not suitable in all types of patients, especially in patients with LA diameter exceeding 48 mm, and extra RFA was often required to achieve PWI [10, 50]. More RCT trials are needed in the future.

### 4.4 Procedural Adverse Events Related to Adjunctive PWI

There was not an increased risk of adverse events related to procedures when adjunctive PWI to PVI compared with PVI alone in our meta-analysis, no matter in safety endpoint analysis or in subgroup analyses. Although atrio-esophageal fistula (AEF) is rare in occurrence rate, it is still taken for serious and fatal complication during and after AF ablation procedures, especially in RFA. A nationwide survey conducted by Gandjbakhch *et al*. [51] reported that the estimated incidence rate may be 25 for 100,000 procedures. All cases of AEF occurred after RFA, no cases were reported after CBA, 63% were seen persistent AF, and 37% of them underwent additional roof or more posterior linear ablation after PVI [51]. The recent meta-analysis revealed that the additional PWI to PVI always gave rise to evidently longer ablation time and total procedural time. But if a careful ablation protocol was followed, PWI seemed not to be relevant to an extra increase of procedure complications [40]. With the rapid development of ablation technology and strategy, the balance between safety and efficacy needs deep consideration and careful discussion.

### 4.5 Future Perspectives

Further studies, like PIVoTAL-IDE (Left Atrial Posterior Wall and PV Isolation Using Cryoballoon for Treatment of Persistent AF(NCT04505163)), STARAF3 (Strategies for Catheter Ablation of peRsistent Atrial Fibrlllation(NCT04428944)), LEAP-AF (Left Atrial Posterior Wall Additional Isolation for Persistent Atrial Fibrillation Trial(NCT04405258)) and HOT (High-density Mapping-guided bOx Isolation and subsTrate Ablation(NCT03998956)) which are currently under way, are needed to verify and elucidate these issues mentioned above. Besides that, the extra ablation strategies for non-PV triggers beyond PVI and PWI is a popular topic with widespread attention for the moment but still remain controversial and ambiguous. Previous study revealed that non-PV triggers can distribute in different areas of both atria like coronary sinus, mitral valve, ligament of Marshall, left atrial appendage, superior vena cava, tricuspid valve besides posterior wall, which may imply that more than one AF trigger coexist in the same patient at the same time [44]. One recent meta-analysis research focus on this issue related to the question which is the best and widely accepted ablation strategy for persistent AF. The study reached the conclusion that the ablation strategy of PVI combined with PWI and non-PV trigger ablation showed the best treatment effect in terms of the primary outcome [52]. Meanwhile, another study also focused on the issue and revealed that enough ablation of non-PV triggers beyond PVI and PWI can decrease the recurrence of AF in patients with special diseases easy to develop AF distinctly [53]. However, guidelines regarding ablation of AF still suggest that non-PV triggers ablation targets remain uncertain, which might be explained by the concern of potential negative conditions, including a higher occurrence rate of complications [2, 54]. More RCTs and meta-analyses can be conduct to compare the effect of the ablation of non-PV triggers and additional anatomic ablation targets, so that we can find the better protocol for catheter ablation of AF patients, especially persistent AF.

### 4.6 Limitations

Several limitations in our analysis must be taken into considerations. Firstly, more than half of studies enrolled in the present analysis are not randomized trials, thus the results are driven predominantly from retrospective analyses and non-randomized studies, which can lead to bias to some extent. Then, the enrolled researches employed different ablation energy and techniques to achieve PWI. Heterogeneities among operators and centers may contribute to another bias. Though our results at last reported the effeciency of PWI in AF ablation both in CBA or RFCA, more RCT trials with comparable levels of operators and definite ablation protocols are needed. Moreover, the methods to monitor arrhythmic events were diverse and heterogenous among the enrolled researches. Part of them merely used electrocardiograph during every follow-up visit, while some others detected the arrhythmia with long-time Holter, or even implantable devices, which might leave an inestimable influence on the results. Finally, follow-up periods, ranging from 6 months to more than 3 years, were significant different in trials included. Aryana *et al*. [18] mentioned the positive results about the effeciency of PWI after a follow-up period of 39 ± 9 months, while there were no meaningful differences at 12 months.

## 5. Conclusions

We conducted a systematical review and meta-analysis to reveal a comprehensive understanding and discussion about the efficiency and safety of additional PWI to PVI among AF patients. In brief, AF patients suitable for catheter ablation, additional PWI to PVI was relevant with decreased postoperative recurrence of AF and atrial arrhythmias compared with PVI alone without an increased incidence rate of AFL or AT, especially in PerAF patients and patients undergoing ablation with cryoballoon. It’s worth noting that adjunctive PWI with radiofrequency ablation may induce a slight increase of recurrent AFL/AT compared with PVI only. More systematic and standardized randomized trials with long-range follow-up period are needed to further explore the therapeutic effect of the adjunctive PWI to PVI in AF patients.

## Data Availability

The data presented in this study are already provided as part of the submitted article and further information can be available on request to the corresponding author (zhpdoc@126.com) for purposes of reproducing the results or replicating the procedure.

## Abbreviations

AF, Atrial fibrillation; PAF, paroxysmal atrial fibrillation; PerAF, persistent atrial fibrillation; LAPWI, left atrial posterior wall isolation; PWI, Posterior wall isolation; PVI, Pulmonary vein isolation; AT, Atrial tachycardia; AFL, Atrial flutter; CBA, cryoballoon ablation; RFA, radiofrequency ablation.

## Supplementary Material

Supplementary material associated with this article can be found, in the online version, at https://doi.org/10.31083/j.rcm2506210.
